# Comparative efficacy of Huatan Jieyu formula versus paroxetine for the treatment of generalized anxiety disorder

**DOI:** 10.1097/MD.0000000000049603

**Published:** 2026-07-10

**Authors:** Fangfang Hu, Zhenzhu Hu, Yue Wu, Qingqing Li, Yanan Xu, Yu Shi

**Affiliations:** a Department of Neurology, Xuzhou Xinjiankang Elderly Disease Hospital, Xuzhou City, Jiangsu Province, China; b Department of Neurology, Xuzhou Cancer Hospital, Xuzhou City, Jiangsu Province, China; c Endocrinology Department of Jiawang District People’s Hospital, Xuzhou City, Jiangsu Province, China; d Department of Neurology, Nanjing Jiangbei Hospital, Nanjing City, Jiangsu Province, China.

**Keywords:** generalized anxiety disorder, Hamilton Anxiety Rating Scale, Huatan Jieyu Formula, paroxetine, Pittsburgh Sleep Quality Index

## Abstract

Generalized anxiety disorder (GAD) is commonly accompanied by depressive symptoms, sleep disturbance, and somatic symptom burden, which may influence treatment selection and real-world outcomes. This retrospective cohort study evaluated the comparative effectiveness and tolerability of Huatan Jieyu Formula (HTJYF) versus paroxetine in adults with GAD treated at a single institution between September 2023 and December 2024. Eligible patients received an 8-week course of HTJYF (n = 38) or paroxetine (n = 40). Anxiety severity was assessed using the Hamilton Anxiety Rating Scale (HAMA) and the Generalized Anxiety Disorder 7-item scale (GAD-7). Secondary measures included the 17-item Hamilton Depression Rating Scale, Pittsburgh Sleep Quality Index, and Patient Health Questionnaire-15. Baseline characteristics were broadly comparable between groups. Both treatments were associated with significant within-group reductions in HAMA and GAD-7 scores from baseline to week 8 (all *P* < .001), while between-group differences in anxiety improvement were not statistically significant (HAMA change: 1.21 points, 95% confidence interval − 0.45 to 2.87, *P* = .151; GAD-7 change: 0.90 points, 95% confidence interval − 0.26 to 2.06, *P* = .125). Improvements in depressive symptoms and somatic symptom burden were similar between groups, whereas sleep quality improved slightly more with HTJYF (Pittsburgh Sleep Quality Index ΔΔ = 0.90, *P* = .045). Treatment-emergent adverse events occurred at comparable rates (35.0% vs 28.9%, *P* = .567). No serious adverse events were observed. Because of the retrospective, nonrandomized design and modest sample size, these findings should be interpreted as hypothesis-generating associations rather than evidence of causal superiority, equivalence, or noninferiority.

## 1. Introduction

Generalized anxiety disorder (GAD) is a prevalent and persistent anxiety condition characterized by excessive and difficult-to-control worry accompanied by autonomic and cognitive symptoms, functional impairment, and frequent psychiatric comorbidity. Contemporary clinical literature emphasizes its substantial population burden and its clinically relevant overlap with depressive symptoms, which together contribute to chronicity and increased healthcare utilization.^[1]^ Beyond core anxiety features, sleep disturbance and insomnia are common in GAD and are associated with greater symptom severity and poorer outcomes. Recent syntheses have quantified a high prevalence of sleep disturbances in GAD and highlighted insomnia-related mechanisms as plausible treatment-relevant pathways.^[2,3]^

Current evidence-based management strategies for GAD include psychological interventions and pharmacotherapy, with selective serotonin reuptake inhibitors (SSRIs) remaining a commonly used first-line medication class in many practice settings.^[4]^ However, treatment effectiveness is heterogeneous; a clinically meaningful subset of patients demonstrate incomplete response, residual symptoms, or treatment resistance, which has motivated interest in alternative or adjunctive approaches that target multidimensional symptom domains. Medication tolerability and acceptability also influence real-world outcomes. A recent network meta-analysis of selective serotonin reuptake inhibitor/serotonin–norepinephrine reuptake inhibitor (SSRI/SNRI) adverse event profiles across anxiety disorders reported differential risks across agents, supporting the clinical importance of adverse-effect monitoring and individualized treatment selection.^[5]^ In addition, inter-individual variability in antidepressant exposure and adverse effects may be partly explained by pharmacogenetic factors; prospective evidence in paroxetine-treated patients has shown clinically relevant associations between Cytochrome P450 2D6 (CYP2D6) activity and safety outcomes, underscoring the need to consider patient-level factors when evaluating comparative effectiveness.^[6]^

Symptom measurement is central to comparative effectiveness research in GAD. In routine clinical practice, anxiety severity is often quantified using clinician-rated and patient-reported scales, while somatic symptom burden and sleep quality are frequently captured using structured instruments. Recent evidence has further clarified the psychometric performance of commonly used somatic symptom measures such as the Patient Health Questionnaire [PHQ-15], supporting their interpretability in clinical studies.^[7]^ In parallel, complementary and integrative interventions for anxiety disorders have received increasing research attention, including acupuncture and traditional Chinese medicine approaches, with recent reviews summarizing evidence for symptom improvement while also noting methodological heterogeneity and the need for comparative studies against standard pharmacotherapy.^[8]^ Against this background, evaluating real-world comparative outcomes between a traditional Chinese medicine-based regimen (Huatan Jieyu Formula [HTJYF]) and an SSRI (paroxetine) may inform pragmatic decision-making regarding symptom reduction, sleep-related outcomes, and tolerability in patients with GAD.

## 2. Materials and methods

### 2.1. Study design

This study was approved by the Ethics Committee of XuZhou Elderly Disease Hospital. This retrospective cohort study consecutively enrolled patients with a clinical diagnosis of GAD who received treatment at our institution between September 2023 and December 2024. A total of 78 eligible cases were included. According to the initial therapeutic regimen documented in the medical records, patients were categorized into an observation group treated with HTJYF (n = 38) and a control group treated with paroxetine (n = 40). Inclusion criteria were as follows: diagnosis of GAD established by qualified psychiatrists in accordance with standardized diagnostic criteria (e.g., Diagnostic and Statistical Manual of Mental Disorders, Fifth Edition [DSM-5] or International Classification of Diseases, Tenth/Eleventh Revision [ICD-10/11] as applied locally); age ≥ 18 years; initiation of either HTJYF or paroxetine as the primary antianxiety treatment during the study period, with no concurrent initiation of other antidepressants, anxiolytics, antipsychotics, mood stabilizers, or additional Chinese herbal formulas intended for anxiolysis; and availability of complete clinical records, including baseline demographic and clinical assessments and follow-up data sufficient for outcome evaluation. Exclusion criteria included: comorbid severe psychiatric disorders (e.g., bipolar disorder, schizophrenia spectrum disorders), high suicide risk, or cognitive impairment precluding valid assessment; primary diagnosis other than GAD (e.g., panic disorder, obsessive–compulsive disorder, post-traumatic stress disorder) when judged to be the main clinical condition; current substance use disorder; severe or unstable medical illnesses (e.g., significant hepatic/renal dysfunction, uncontrolled cardiovascular or endocrine disease) that could confound treatment response or compromise safety; pregnancy or lactation; known allergy or intolerance to components of HTJYF or to paroxetine; and substantial missing data or documented poor adherence that prevented reliable analysis. The study was conducted in accordance with the Declaration of Helsinki and was approved by the institutional medical ethics committee; written informed consent was obtained from all participants prior to inclusion.

### 2.2. Treatment protocol and concomitant therapy

The treatment protocol comprised an 8-week intervention period. In the observation group, participants received Huatan Jieyu Granules at a dose of 1 sachet (15 g) twice daily; each dose was dissolved in warm water and administered orally in the morning and evening after meals. In the control group, participants received paroxetine hydrochloride tablets at a dose of 1 tablet once daily, taken orally in the morning. Concomitant therapies were permitted under predefined conditions: for participants with clinically significant insomnia, (es)zopiclone could be administered once nightly at bedtime at a conventional therapeutic dose, not exceeding the maximum dose indicated in the product labeling for insomnia; to minimize potential acute sedative effects on psychometric outcomes, all scale-based assessments were scheduled at least 12 hours after the administration of sedative–hypnotic agents. The cumulative duration of sedative–hypnotic use during the trial was restricted to no more than 2 weeks. Ongoing medications for stable comorbid chronic conditions (e.g., antihypertensive or antidiabetic agents) were allowed without modification whenever clinically feasible. Except for the prespecified permitted concomitant treatments, no additional pharmacologic therapies targeting anxiety disorders or somatization-related symptoms were allowed during the study period. No individualized modification of the HTJYF prescription was allowed during the 8-week observation period.

### 2.3. Composition, preparation, and quality control of HTJYF

HTJYF was used as a fixed granule prescription in this cohort. The formula contained the following herbal components: Coptidis Rhizoma (Huanglian), Citri Reticulatae Pericarpium (Chenpi), Poria (Fuling), Glycyrrhizae Radix et Rhizoma (Gancao), Aurantii Fructus Immaturus (Zhishi), Bambusae Caulis in Taenias (Zhuru), Polygoni Multiflori Caulis (Shouwuteng), Platycladi Semen (Baiziren), Ziziphi Spinosae Semen (Suanzaoren), Chuanxiong Rhizoma (Chuanxiong), and Acori Tatarinowii Rhizoma (Shichangpu). Each dispensed sachet contained 15 g of mixed granules, and the daily dose was 30 g. The same fixed prescription and dosing schedule were used for all patients included in the HTJYF group.

The granules were prepared and dispensed through the institutional pharmacy under standardized operating procedures. Briefly, qualified herbal materials or concentrated single-herb granules were weighed according to the fixed prescription, mixed uniformly, packaged into 15-g sachets, and labeled with the product name, dose, batch number, dispensing date, expiration date, and storage requirements. Patients were instructed to dissolve 1 sachet in warm water and take it after meals twice daily.

Quality control was implemented before clinical dispensing. The institutional pharmacy verified supplier qualification, batch traceability, prescription consistency, package integrity, and expiration date. Only batches that met the predefined quality requirements were dispensed to study patients, and batch numbers were retained in the medication records to support traceability.

### 2.4. Data collection

Data were retrospectively extracted from the institutional electronic medical record system for eligible patients treated between September 2023 and December 2024 using a standardized case-report form. Baseline variables included demographics (age, sex), disease characteristics (duration, first episode/relapse, prior treatment history), symptom severity scales recorded in routine care (Hamilton Anxiety Rating Scale [HAMA], Generalized Anxiety Disorder 7-item scale [GAD-7], and where available 17-item Hamilton Depression Rating Scale [HAMD-17], Pittsburgh Sleep Quality Index [PSQI], and PHQ-15), comorbidities (e.g., hypertension, diabetes mellitus), and concomitant chronic medications. Treatment exposure data comprised the initial regimen (HTYJF vs paroxetine), prescribed dose and schedule, treatment duration, and permitted concomitant sedative–hypnotic use for insomnia (including dose and cumulative days). Outcomes were collected at baseline and week 8 (or the closest assessment within the week 8 window), including continuous scale scores and derived categorical endpoints (HAMA-based response ≥ 50% reduction; remission defined as week 8 HAMA ≤ 7). Safety data included treatment-emergent adverse events (TEAEs) documented during the 8-week period, categorized by major symptom/system organ class, and treatment modification or discontinuation with recorded reasons.

Because this was a retrospective cohort based on all eligible cases available during the study period, no a priori sample size calculation was performed. To clarify the implications of sample size, we performed a post hoc power analysis for the 2 primary continuous between-group comparisons using a 2-sided independent-samples *t*-test, α = 0.05, the observed group sizes (n = 40 and n = 38), and the observed standardized between-group differences in change scores. The minimum detectable standardized difference with approximately 80% power was also estimated to aid interpretation.

### 2.5. Statistical analysis

All statistical analyses were performed using IBM SPSS Statistics, version 26.0 (IBM Corp., Armonk). Continuous variables were summarized as mean ± standard deviation and categorical variables as number (percentage). Baseline characteristics were compared between groups using the independent-samples *t*-test for continuous variables and the chi-square test or Fisher’s exact test for categorical variables, as appropriate. Within-group changes from baseline to week 8 were assessed using paired-samples *t*-tests. Between-group differences in change scores (week 8 minus baseline) were evaluated using independent-samples *t*-tests. Categorical efficacy endpoints, including response (≥50% reduction in HAMA from baseline) and remission (week 8 HAMA ≤ 7), were compared using the chi-square test or Fisher’s exact test, and effect estimates were expressed as risk ratios with 95% confidence intervals (CIs). All tests were 2-sided, and *P* < .05 was considered statistically significant.

## 3. Results

### 3.1. Baseline characteristics and between-group comparability

Baseline demographic, clinical, and symptom severity characteristics were broadly comparable between the control group (paroxetine, n = 40) and the observation group (HTYJF, n = 38; Table 1). There were no statistically significant between-group differences in age (39.7 ± 9.4 vs 40.4 ± 6.7 years; *t* = −0.38, *P* = .705) or disease duration (3.26 ± 1.12 vs 3.29 ± 1.29 years; *t* = −0.11, *P* = .913). Sex distribution was similar (male: 40.0% vs 26.3%; χ^2^ = 1.64, *P* = .200). The proportion of first episode GAD was numerically lower in the observation group (75.0% vs 55.3%), but the difference did not reach statistical significance (χ^2^ = 3.35, *P* = .067). Prior treatment history was comparable between groups (52.5% vs 50.0%; χ^2^ = 0.05, *P* = .825). Baseline anxiety severity did not differ between groups as assessed by HAMA (22.84 ± 4.33 vs 23.65 ± 3.75; *t* = −0.88, *P* = .379) and GAD-7 (14.15 ± 3.26 vs 14.90 ± 2.44; *t* = −1.15, *P* = .252), and depressive symptoms measured by HAMD-17 were also similar (13.61 ± 3.94 vs 12.79 ± 4.86; *t* = 0.82, *P* = .417). Sleep quality (PSQI) and baseline insomnia complaints were comparable (PSQI: 9.44 ± 2.74 vs 9.67 ± 2.75, *t* = −0.37, *P* = .713; insomnia complaint: 47.5% vs 47.4%, χ^2^ < 0.01, *P* = .991). Somatic symptom burden (PHQ-15) was numerically higher in the observation group (11.31 ± 3.47 vs 12.72 ± 3.52), without statistical significance (*t* = −1.78, *P* = .079). The prevalence of hypertension and diabetes mellitus, as well as concomitant antihypertensive and antidiabetic medication use, did not differ between groups (all *P* > .05). Overall, effect size estimates indicated acceptable baseline balance (maximum SMD = 0.40; maximum Cramer’s *V* = 0.21).

**Table 1:** Baseline characteristics and between-group comparisons.

Variable	Control (Paroxetine; n = 40)	Observation (Huatan Jieyu Formula; n = 38)	Test statistic	*P* value	Effect size
Age, yr	39.7 ± 9.4	40.4 ± 6.7	*t* = −0.38	.705	SMD = 0.09
Disease duration, yr	3.26 ± 1.12	3.29 ± 1.29	*t* = −0.11	.913	SMD = 0.02
HAMA score	22.84 ± 4.33	23.65 ± 3.75	*t* = −0.88	.379	SMD = 0.20
GAD-7 score	14.15 ± 3.26	14.90 ± 2.44	*t* = −1.15	.252	SMD = 0.26
HAMD-17 score	13.61 ± 3.94	12.79 ± 4.86	*t* = 0.82	.417	SMD = 0.19
PSQI score	9.44 ± 2.74	9.67 ± 2.75	*t* = −0.37	.713	SMD = 0.08
PHQ-15 score	11.31 ± 3.47	12.72 ± 3.52	*t* = −1.78	.079	SMD = 0.40
Male sex, n (%)	16 (40.0)	10 (26.3)	χ^2^ = 1.64	.200	*V* = 0.15
First episode, n (%)	30 (75.0)	21 (55.3)	χ^2^ = 3.35	.067	*V* = 0.21
Prior treatment history, n (%)	21 (52.5)	19 (50.0)	χ^2^ = 0.05	.825	*V* = 0.02
Insomnia complaint at baseline, n (%)	19 (47.5)	18 (47.4)	χ^2^ < 0.01	0.991	*V* < 0.01
Hypertension, n (%)	6 (15.0)	8 (21.1)	χ^2^ = 0.48	.486	*V* = 0.08
Diabetes mellitus, n (%)	5 (12.5)	7 (18.4)	χ^2^ = 0.52	.469	*V* = 0.08
Concomitant antihypertensive medication, n (%)	4 (10.0)	7 (18.4)	χ^2^ = 1.14	.285	*V* = 0.12
Concomitant antidiabetic medication, n (%)	5 (12.5)	8 (21.1)	χ^2^ = 1.03	.311	*V* = 0.11

GAD = generalized anxiety disorder, GAD-7 = Generalized Anxiety Disorder 7-item scale, HAMA = Hamilton Anxiety Rating Scale, HAMD-17 = 17-item Hamilton Depression Rating Scale, PSQI = Pittsburgh Sleep Quality Index, PHQ-15 = Patient Health Questionnaire-15, SMD = standardized mean difference (Cohen’s *d*, absolute value), *V* = Cramer’s *V*.

### 3.2. Primary outcomes at week 8

In the cohort, both treatment groups showed marked reductions in anxiety severity from baseline to week 8 (Table 2). Within-group changes indicated statistically significant improvement on both the HAMA and GAD-7 in the control and observation groups (all paired comparisons *P* < .001). However, between-group comparisons demonstrated no statistically significant differences in treatment effect: the between-group difference in mean HAMA change (observation minus control) was 1.21 points (95% CI, −0.45 to 2.87; Welch *t* = 1.451; *P* = .151), and the between-group difference in mean GAD-7 change was 0.90 points (95% CI, −0.26 to 2.06; Welch *t* = 1.550; *P* = .125). Consistent with these findings, response and remission rates at week 8 (HAMA-based definitions) were numerically higher in the observation group but did not differ significantly between groups (response: Fisher *P* = .602; remission: Fisher *P* = .268).

**Table 2:** Primary outcomes at baseline and week 8, change scores, and categorical efficacy endpoints.

Outcome	Control (Paroxetine; n = 40)	Observation (Huatan Jieyu Formula; n = 38)	Between-group comparison
HAMA score	22.84 ± 4.33 → 10.10 ± 3.80; Δ 12.74 (95% CI, 11.57–13.91)	23.65 ± 3.75 → 9.70 ± 3.70; Δ 13.95 (95% CI, 12.73–15.17)	ΔΔ 1.21 (95% CI, −0.45 to 2.87); Welch *t* = 1.451; *P* = .151
GAD-7 score	14.15 ± 3.26 → 6.45 ± 2.35; Δ 7.70 (95% CI, 6.83–8.57)	14.90 ± 2.44 → 6.30 ± 2.40; Δ 8.60 (95% CI, 7.81–9.39)	ΔΔ 0.90 (95% CI, −0.26 to 2.06); Welch *t* = 1.550; *P* = .125
Response at week 8, n (%)	29 (72.5)	30 (78.9)	RR = 1.09 (95% CI, 0.85–1.40); Fisher *P* = .602
Remission at week 8, n (%)	6 (15.0)	10 (26.3)	RR = 1.75 (95% CI, 0.71–4.36); Fisher *P* = .268

CI = confidence interval, GAD-7 = Generalized Anxiety Disorder 7-item scale, HAMA = Hamilton Anxiety Rating Scale, RR = risk ratio, Δ = within-group mean change (baseline minus week 8), ΔΔ = between-group difference in mean change (observation minus control).

### 3.3. Secondary outcomes

Both groups exhibited improvements in depressive symptoms, sleep quality, and somatic symptom burden from baseline to week 8 (Table 3). Within-group analyses showed significant reductions in HAMD-17, PSQI, and PHQ-15 scores in both the control and observation groups (all paired comparisons *P* < .001). Between-group comparisons indicated that improvements in depressive symptoms (HAMD-17) and somatic symptom burden (PHQ-15) were comparable (HAMD-17: ΔΔ = 0.20, 95% CI, −1.20 to 1.60; Welch *t* = 0.28, *P* = .777; PHQ-15: ΔΔ = 0.30, 95% CI, −0.94 to 1.54; Welch *t* = 0.48, *P* = .632). For sleep quality, the observation group demonstrated a slightly greater reduction in PSQI compared with the control group (ΔΔ = 0.90, 95% CI, 0.02 to 1.78; Welch *t* = 2.04, *P* = .045). Among participants reporting insomnia at baseline, insomnia resolution rates at week 8 did not differ significantly between groups (63.2% vs 72.2%; Fisher’s exact *P* = .728).

**Table 3:** Secondary outcomes at baseline and week 8.

Outcome	Control (Paroxetine; n = 40)	Observation (Huatan Jieyu Formula; n = 38)	Between-group comparison
HAMD-17 score	13.61 ± 3.94 → 8.41 ± 3.60; Δ 5.20 (95% CI, 4.24–6.16)	12.79 ± 4.86 → 7.39 ± 4.00; Δ 5.40 (95% CI, 4.35–6.45)	ΔΔ 0.20 (95% CI, −1.20 to 1.60); Welch *t* = 0.28; *P* = .777
PSQI score	9.44 ± 2.74 → 7.24 ± 2.30; Δ 2.20 (95% CI, 1.59–2.81)	9.67 ± 2.75 → 6.57 ± 2.40; Δ 3.10 (95% CI, 2.44–3.76)	ΔΔ 0.90 (95% CI, 0.02 to 1.78); Welch *t* = 2.04; *P* = .045
PHQ-15 score	11.31 ± 3.47 → 8.51 ± 3.10; Δ 2.80 (95% CI, 1.94–3.66)	12.72 ± 3.52 → 9.62 ± 3.20; Δ 3.10 (95% CI, 2.18–4.02)	ΔΔ 0.30 (95% CI, −0.94 to 1.54); Welch *t* = 0.48; *P* = .632
Insomnia resolution at week 8, n/N (%)	12/19 (63.2)	13/18 (72.2)	Fisher *P* = .728

CI = confidence interval, HAMD-17 = 17-item Hamilton Depression Rating Scale, PHQ-15 = Patient Health Questionnaire-15, PSQI = Pittsburgh Sleep Quality Index, Δ = within-group mean change (baseline minus week 8), ΔΔ = between-group difference in mean change (observation minus control).

### 3.4. Safety and tolerability

Overall, the incidence of TEAEs over 8 weeks was comparable between the control (paroxetine) and observation (HTYJF) groups (35.0% vs 28.9%; χ^2^ = 0.33, *P* = .567; Table 4). Gastrointestinal and nervous system events were the most frequently reported TEAE categories, without significant between-group differences (gastrointestinal disorders: 15.0% vs 13.2%, χ^2^ = 0.05, *P* = .815; nervous system disorders: 12.5% vs 10.5%, Fisher *P* = 1.000). Sexual dysfunction occurred more frequently in the control group (15.0% vs 0.0%; Fisher *P* = .026). No serious adverse events or deaths were recorded in either group. TEAE-related treatment discontinuation was infrequent and comparable between groups (5.0% vs 2.6%; Fisher *P* = 1.000), and dose reduction or temporary interruption was also uncommon (7.5% vs 2.6%; Fisher *P* = .616). Overall treatment discontinuation for any reason occurred in 7.5% (3/40) of control patients and 5.3% (2/38) of observation patients (Fisher *P* = 1.000). TEAE-related discontinuations were attributed to nausea (n = 1) and somnolence (n = 1) in the control group and to skin intolerance symptoms (n = 1) in the observation group; discontinuations due to insufficient efficacy were rare (1/40 vs 1/38; Fisher *P* = 1.000).

**Table 4:** Safety and tolerability outcomes during the 8-week treatment period.

Event (patients with ≥1 event)	Control (Paroxetine) n = 40, n (%)	Observation (Huatan Jieyu Formula) n = 38, n (%)	Test statistic	*P* value
Any TEAE	14 (35.0)	11 (28.9)	χ^2^ = 0.33	.567
Gastrointestinal disorders	6 (15.0)	5 (13.2)	χ^2^ = 0.05	.815
Nausea	4 (10.0)	2 (5.3)	Fisher	.676
Nervous system disorders	5 (12.5)	4 (10.5)	Fisher	1.000
Somnolence	3 (7.5)	1 (2.6)	Fisher	.616
Dry mouth	4 (10.0)	1 (2.6)	Fisher	.359
Sexual dysfunction	6 (15.0)	0 (0.0)	Fisher	.026
Discontinuation due to TEAE	2 (5.0)	1 (2.6)	Fisher	1.000
Dose reduction/temporary interruption	3 (7.5)	1 (2.6)	Fisher	.616
Treatment discontinuation (any reason)	3 (7.5)	2 (5.3)	Fisher	1.000

TEAE = treatment-emergent adverse event.

## 4. Discussion

In this retrospective cohort comparing HTYJF and paroxetine over an 8-week treatment period in patients with GAD, both groups demonstrated substantial reductions in anxiety severity from baseline to week 8, as reflected by statistically significant within-group improvements on the clinician-rated HAMA and the patient-reported GAD-7. The absence of statistically significant between-group differences in the mean change scores for both HAMA and GAD-7 suggests that, within the constraints of the present design and sample size, the anxiolytic effectiveness of HTYJF was comparable to that of paroxetine. This finding aligns with contemporary guidance that SSRIs constitute a recommended first-line pharmacological option for moderate to severe GAD, while also underscoring that alternative strategies may achieve similar short-term symptom reduction in routine practice settings.^[9,10]^

Several considerations may explain why the between-group differences in anxiety improvement were not statistically significant despite numerically greater reductions in the observation group. First, the study may have been underpowered to detect small-to-moderate differences in continuous outcomes, particularly when variability in real-world response trajectories is expected. Second, regression toward the mean and nonspecific therapeutic effects (e.g., clinical contact, expectancy) may contribute to symptomatic improvement in both groups, thereby attenuating between-group contrasts. Third, the permitted short-term use of hypnotics for severe insomnia (within predefined limits) could indirectly influence perceived anxiety severity, sleep-related items, and overall well-being, reducing the ability to attribute changes exclusively to the index treatments. Finally, because this was not a randomized or blinded comparison, baseline differences not fully captured by measured covariates (e.g., symptom chronicity, psychosocial stressors, treatment preference) may have influenced treatment selection and outcomes, potentially biasing treatment effect estimates toward equivalence. Beyond symptom score changes, response and remission rates defined by HAMA thresholds were numerically higher in the Huatan Jieyu group but did not differ significantly from the paroxetine group. Clinically, this pattern is consistent with a scenario in which both interventions yield meaningful symptomatic improvement for many patients, while a smaller subset achieves near-complete resolution within 8 weeks. The lack of statistically significant differences in categorical endpoints may also reflect limited event counts and the inherent information loss when continuous outcomes are dichotomized; therefore, the continuous change scores likely provide a more sensitive summary of comparative short-term benefit.

Regarding secondary outcomes, both groups improved in depressive symptoms (HAMD-17), sleep quality (PSQI), and somatic symptom burden (PHQ-15), consistent with the high frequency of affective symptoms, sleep disturbance, and somatization in GAD populations. Between-group comparisons showed broadly comparable improvements in depressive and somatic symptom measures, while the Huatan Jieyu group had a slightly greater reduction in PSQI. Although the PSQI between-group difference reached nominal statistical significance (*P* = .045), the magnitude of the difference was modest and should be interpreted with caution. Multiple secondary outcomes increase the probability of type I error if formal multiplicity adjustment is not applied, and sleep outcomes may be particularly sensitive to concomitant hypnotic exposure, baseline insomnia severity, and differential reporting. In addition, insomnia resolution rates among those reporting insomnia at baseline did not differ significantly between groups, which further supports interpreting the PSQI signal as a small effect that warrants prospective confirmation rather than a definitive comparative advantage. Safety findings were clinically relevant. Overall TEAE incidence was similar across groups, and no serious adverse events occurred. Importantly, sexual dysfunction was more frequent in the paroxetine group, consistent with the established adverse-effect profile of SSRIs and pharmacovigilance/clinical literature indicating that antidepressant-associated sexual dysfunction is common and may differ by agent, with paroxetine often reported among those associated with higher risk.^[11,12]^ The observation of fewer sexual adverse events with HTYJF may therefore represent a clinically meaningful tolerability consideration for shared decision-making, particularly for patients for whom sexual functioning is a priority or those who have previously discontinued SSRIs due to sexual side effects. Nevertheless, adverse event ascertainment in retrospective settings is frequently incomplete, and differential reporting between herbal and conventional medications is plausible; accordingly, prospective standardized safety monitoring remains necessary.

The present findings of broadly comparable anxiolytic improvement between a Chinese herbal formula and conventional pharmacotherapy are consistent with several recent syntheses and guideline documents, although the evidence base remains heterogeneous. A 2025 systematic review and meta-analysis of Chinese herbal medicine (CHM) for anxiety disorders reported that, compared with anxiolytic medication, CHM was associated with a modest reduction in HAMA scores in GAD and fewer adverse events, including in analyses restricted to trials judged at low risk of bias.^[13]^ While the comparator class in that review primarily involved anxiolytics (often benzodiazepines) rather than SSRIs, the direction of effect supports the plausibility that CHM can achieve clinically relevant symptom reductions with a potentially favorable tolerability profile in some settings. In parallel, a 2023 evidence synthesis focusing on Xiao Yao San (XYS) concluded that XYS combined with anxiolytics appeared safe and effective for anxiety disorders, while emphasizing the need for larger, methodologically robust trials to clarify the effectiveness and adverse events of XYS monotherapy.^[14]^ Although XYS and HTYJF are distinct interventions, these findings collectively suggest that herbal formulations may contribute to symptom relief and may be used either as stand-alone or adjunctive strategies, with the important caveat that formula composition, dosing standardization, and study quality materially influence generalizability. From a conventional treatment perspective, a 2022 clinical practice guideline recommends SSRIs as first-line pharmacological therapy for moderate GAD not improved with educational/self-management interventions and for severe cases pending specialist assessment. Paroxetine remains an approved option for GAD, with labeling describing evidence from 8-week placebo-controlled trials and recommending 20 mg daily as a typical starting and recommended dose, with titration based on response and tolerability.^[15]^ Together, recent syntheses and guidance support SSRIs as standard therapy while leaving open a role for herbal options – particularly where tolerability, patient preference, or access considerations are prominent – provided that safety and product quality are assured.

However, several limitations should be emphasized. First, the retrospective, nonrandomized design is inherently susceptible to confounding by indication, selection bias, information bias, and incomplete documentation. Treatment choice may have been influenced by clinician judgment, patient preference, expected tolerability, previous medication experience, symptom profile, or unrecorded socioeconomic and psychosocial factors; these variables could not be fully controlled using the available routine-care data. Second, outcome measurement and adverse event ascertainment were based on medical records, which may be less standardized than prospective trial assessments and may underestimate mild or sensitive adverse events. Third, although sedative–hypnotic use was restricted, its availability for clinically significant insomnia may still have affected sleep-related outcomes and daytime symptom ratings. Fourth, the modest sample size limited the statistical power to detect small-to-moderate between-group differences, restricted the precision of effect estimates, and precluded robust subgroup or adjusted analyses. Therefore, the nonsignificant primary comparisons should be interpreted as inconclusive rather than as evidence of equivalence or noninferiority. Fifth, this was a single-center study with an 8-week follow-up period, limiting the generalizability and durability of the findings. Sixth, although we have described the composition, preparation, and quality control procedures of HTJYF, batch-to-batch variability and differences in herbal granule standards across institutions may affect external validity. Future prospective, randomized, blinded or assessor-blinded controlled trials with prespecified sample size calculations, standardized HTJYF production and quality testing, systematic safety monitoring, longer follow-up, and appropriate control for concomitant hypnotic use are needed to verify these preliminary observations.

## 5. Conclusion

Over 8 weeks, HTYJF and paroxetine were both associated with significant improvements in anxiety severity and related symptoms, with no statistically significant between-group differences in primary efficacy outcomes. Secondary outcomes were generally comparable, with a modest advantage for HTYJF in sleep quality. Overall TEAE rates were similar, while sexual dysfunction occurred more frequently with paroxetine. Because of the retrospective, nonrandomized design, modest sample size, and potential residual confounding, these findings should be regarded as hypothesis-generating and should not be interpreted as establishing causal efficacy, superiority, equivalence, or noninferiority. Adequately powered prospective randomized controlled trials with standardized HTJYF quality control are required to confirm the comparative efficacy and safety of HTJYF for GAD.

## Author contributions

**Conceptualization:** Fangfang Hu, Zhenzhu Hu, Yue Wu, Qingqing Li, Yanan Xu, Yu Shi.

**Data curation:** Fangfang Hu, Zhenzhu Hu, Yue Wu, Qingqing Li, Yanan Xu, Yu Shi.

**Formal analysis:** Fangfang Hu, Zhenzhu Hu, Yue Wu, Qingqing Li, Yanan Xu, Yu Shi.

**Funding acquisition:** Fangfang Hu, Yanan Xu, Yu Shi.

**Investigation:** Fangfang Hu, Yu Shi.

**Writing – original draft:** Fangfang Hu, Yu Shi.

**Writing – review & editing:** Fangfang Hu, Yu Shi.

## Correction

This article was originally published without funding information. The online version has now been updated with a funding statement text: “This study was supported by the Xuzhou Science and Technology Program (Grant No. KC23236, awarded to H.F.F.), Department of Neurology, Xuzhou New Healthy Geriatric Hospital, Xuzhou, Jiangsu, China; the Xuzhou Science and Technology Program (Grant No. KC23232, awarded to S.Y.), Department of Neurology, Xuzhou Cancer Hospital, Xuzhou, Jiangsu, China; and the Project of the Jiangsu Provincial Health Commission (Grant No. Z2024012, awarded to S.Y.), Department of Neurology, Xuzhou Cancer Hospital, Xuzhou, Jiangsu, China.”
